# Anonymization of Portuguese Clinical Notes Using Large Language Models and Quantum-Enhanced Hybrid Architectures: Comparative Evaluation Study

**DOI:** 10.2196/91513

**Published:** 2026-09-14

**Authors:** Samer Rahmeh, Felipe Meneguitti Dias, Ramon Alfredo Moreno, Marina De Fatima De Sa Rebelo, Daniela Herrmann, Jose Eduardo Krieger, Marco Antonio Gutierrez

**Affiliations:** 1 Quantum Solutions Architecture Dept, Q.Enterprises AG (Dynex), Baar, Zug, Switzerland; 2 Instituto do Coração, Hospital das Clínicas HCFMUSP, Faculdade de Medicina, Universidade de São Paulo, Av. Dr. Enéas de Carvalho Aguiar, 44, Sao Paulo, São Paulo, 05403-000, Brazil, +551126615441

**Keywords:** electronic health record, medical text anonymization, large language models, quantum machine learning, natural language processing, data privacy, HIPAA compliance, GDPR compliance, LGPD compliance, LLMs, QML, NLP

## Abstract

**Background:**

The widespread adoption of electronic health records (EHRs) has generated large-scale repositories of highly sensitive clinical information, emphasizing the need for robust anonymization strategies to enable secondary use for research while safeguarding patient privacy. Conventional rule-based and machine learning approaches for deidentifying medical text face limitations with the linguistic complexity, variability, and context dependence inherent to clinical documentation. Recent advances in large language models (LLMs), combined with emerging quantum computing paradigms, present novel opportunities to enhance the accuracy, scalability, and resilience of health care data anonymization.

**Objective:**

This study aims to evaluate the efficacy of LLM-based and quantum-enhanced hybrid architectures for medical text anonymization, assessing the effectiveness and computational efficiency across multiple entity types in Portuguese clinical notes.

**Methods:**

We constructed a gold-standard corpus of 1000 Portuguese outpatient clinical notes, manually annotated by 5 trained researchers for 5 protected-entity categories: patient names, dates, identifiers, organizations, and geographic locations. Four anonymization strategies were evaluated: 2 stand-alone LLMs (Llama-3.1-8B-instruct and Llama-3.3-70B-instruct) and 2 quantum-enhanced hybrid models (Dynex-QML with 8B and 70B base models) incorporating quantum optimization via Quadratic Unconstrained Binary Optimization (QUBO) formulations. The quantum-enhanced approach transforms the final attention layer of the LLM into a global constraint satisfaction problem solved via neuromorphic quantum annealing. Model performance was measured on a held-out test set of 500 notes using precision, recall, and *F*_1_-score metrics. Computational efficiency was quantified through end-to-end processing time.

**Results:**

The quantum-enhanced Dynex-QML-70B model achieved the highest overall performance with a macro*–F*_1_-score of 0.855 (95% CI 0.823‐0.880), outperforming the stand-alone Llama-3.3-70B (0.726, 95% CI 0.704‐0.747), Dynex-QML-8B (0.733, 95% CI 0.709‐0.756), and Llama-3.1-8B (0.602, 95% CI 0.588‐0.615). Compared with Llama 3.3 70B, Dynex-QML (Llama 70B) improved macro*–F*_1_-score by 0.128 (95% CI 0.091‐0.163; empirical 2-sided bootstrap *P*<.001). Most entity-level within-size comparisons favored the Dynex-QML models and were statistically significant, although the ORGANIZATION comparison between Dynex-QML (Llama 8B) and Llama 3.1 8B was not significant. For total elapsed time, Dynex-QML (Llama 70B) was faster than stand-alone Llama 3.3 70B (7.97, 95% CI 7.72‐8.21 seconds per note vs 8.52, 95% CI 8.23‐8.81 seconds per note). In a paired note-level bootstrap comparison, this corresponded to a mean reduction of 0.55 (SD 2.36; 95% CI −0.75 to −0.34 seconds per note; *P*<.001).

**Conclusions:**

Quantum-enhanced hybrid architectures provide substantial improvements in medical text anonymization accuracy compared to stand-alone LLMs, particularly reductions in false-positive rates while preserving high sensitivity. The Dynex-QML-70B model achieved the best balance between performance and efficiency, suggesting that quantum-enhanced optimization offers a strategy for high-fidelity, scalable, and regulation-compliant deidentification of clinical text. These findings highlight the potential of emerging quantum-AI paradigms to advance the secondary secure use of health care data.

## Introduction

### Background

The widespread adoption of electronic health records (EHRs) has fundamentally transformed how patient information is generated, stored, and accessed in health care systems worldwide [1,2]. These digital repositories enable comprehensive documentation of medical histories, clinical notes, diagnostic results, and other sensitive data, frequently captured in unstructured textual formats [3]. Safeguarding this information is critical not only to preserve patient privacy and confidentiality but also to ensure the integrity and trustworthiness of modern health care systems [4,5].

Core principles of medical data governance, including privacy, confidentiality, integrity, and nonrepudiation, guide how patient information is accessed, shared, and protected [6]. Anonymization has thus become a vital requirement, enabling secondary uses of health data for research and quality improvement while minimizing the risk of patient reidentification [7,8]. The challenge of effective anonymization is particularly acute for unstructured clinical text, which contains complex linguistic patterns, domain-specific terminology, and contextually embedded sensitive information [9,10].

To address these concerns, comprehensive data protection regulations have been established worldwide. In the United States, the Health Insurance Portability and Accountability Act (HIPAA) mandates the deidentification of Protected Health Information (PHI) before secondary use, specifying either Safe Harbor or Expert Determination methods for compliance [11,12]. The European General Data Protection Regulation (GDPR) sets rigorous standards for processing health data, emphasizing techniques such as anonymization and pseudonymization with stringent consent requirements [13,14]. Similarly, Brazil’s Lei Geral de Proteção de Dados Pessoais (LGPD) aligns with international norms, underscoring the need for robust technical solutions to protect sensitive information in the Brazilian health care context [15].

Over time, various anonymization methods have been developed to address these regulatory requirements [16]. Conventional rule-based and pattern-matching techniques, though reliable for structured identifiers such as social security numbers and standardized date formats, often struggle with the linguistic complexity, variability, and context dependence of natural language in medical texts [17,18]. These approaches typically require extensive manual rule creation and maintenance, limiting their scalability across different health care institutions and documentation styles.

Machine learning and deep learning models, particularly those developed for Named Entity Recognition (NER), offer greater adaptability and accuracy in identifying sensitive entities within clinical narratives [19-21]. Transformer-based architectures, including BERT (Bidirectional Encoder Representations from Transformers) and specialized biomedical variants such as BioBERT and ClinicalBERT, have demonstrated strong performance across multiple deidentification benchmarks by effectively capturing contextual and domain-specific linguistic patterns [22,23]. However, these approaches require large annotated corpora, extensive hyperparameter tuning, and considerable computational resources for both training and inference [24].

Most recently, large language models (LLMs) such as GPT-4, Llama, and their instruction-tuned derivatives have emerged as promising alternatives for anonymizing medical texts [25,26]. These models can capture nuanced context, understand complex semantic relationships, and identify entities that may elude traditional pattern-matching systems [27]. Evidence suggests that locally deployable LLMs can achieve competitive performance with state-of-the-art NER systems while offering flexibility in entity categories through prompt engineering [28,29]. Nevertheless, LLM-based approaches remain at a nascent stage in the clinical deidentification literature, with persistent concerns regarding model interpretability, computational overhead, hallucination phenomena, and potential risks when processing sensitive data through external services [30].

Even more exploratory are efforts in quantum computing paradigms, which have begun to investigate whether quantum algorithms can be applied to tasks such as pattern recognition, optimization, and privacy-preserving computation [31,32]. Quantum Natural Language Processing (QNLP) represents an emerging field that leverages quantum mechanical principles for linguistic tasks, with theoretical foundations suggesting potential advantages in expressiveness and computational efficiency [33,34]. The application of quantum machine learning to text processing has been explored through various frameworks, including the translation of classical operations into Quadratic Unconstrained Binary Optimization (QUBO) problems suitable for quantum annealing [35,36]. Neuromorphic quantum computing platforms have emerged that emulate quantum behavior through memristor-based circuits, enabling practical deployment of quantum-inspired algorithms on classical hardware [37]. Despite growing interest, the practical application of quantum-enhanced approaches to clinical machine learning remains largely unexplored, and no systematic evaluations have compared quantum-assisted architectures with contemporary LLM-based or transformer-based methods for clinical text anonymization. The aim of this study is to evaluate the effectiveness and computational efficiency of LLM-based and quantum-enhanced hybrid anonymization architectures on Portuguese clinical notes from a single Brazilian tertiary cardiology hospital, across 5 protected health information entity categories. We hypothesized that integrating a global QUBO-based optimization layer over the final attention layer of an instruction-tuned LLM would yield higher anonymization quality, particularly in precision, than the same LLM operating in stand-alone prompt-based mode, while remaining computationally tractable for routine de-identification workflows.

### Related Works and Our Contribution

Recent studies have explored the use of LLMs for clinical text deidentification. Wiest et al [28] developed an open-source anonymization framework based on locally deployable LLMs, demonstrating that models such as Llama-3-70B can achieve sensitivity exceeding 0.992 on German clinical letters. Their work emphasized 2 critical aspects: the importance of local deployment for privacy preservation and the flexibility of prompt-based approaches for customizing entity definitions. Similar findings have been reported for English clinical texts, where instruction-tuned LLMs have achieved competitive performance against fine-tuned NER systems [26].

Systematic reviews of deidentification approaches have documented the evolution from rule-based systems to hybrid machine learning methods, with transformer-based models representing the current state of the art [19,38]. The 2014 i2b2-UTHealth corpus remains the most frequently used benchmark, where top-performing systems achieve a micro-averaged *F*_1_-score of 0.940 [39]. However, most benchmarks focus on English texts, with limited evaluation of methods for other languages, including Portuguese.

In the quantum computing domain, research in QNLP has demonstrated theoretical advantages for text classification and semantic analysis tasks [33,40]. Hybrid quantum-classical approaches have been proposed that integrate quantum circuit optimization with classical neural network components [41]. However, to our knowledge, no prior work has specifically evaluated quantum-enhanced architectures for medical text anonymization.

In this context, this study makes several contributions. First, we present a comparative evaluation of LLM-based and quantum-enhanced hybrid architectures for medical text anonymization using a purpose-built corpus of Portuguese clinical notes. Second, we demonstrate the application of neuromorphic quantum optimization algorithms to improve anonymization precision while maintaining high recall. Third, we provide a detailed explanation of how transformer attention mechanisms can be reformulated as QUBO problems and solved via quantum annealing. Fourth, we provide a comprehensive assessment of both quality metrics and computational efficiency. Finally, we discuss the implications of our findings for regulatory compliance and practical deployment in health care settings.

The primary aim of this study was to perform a comparative evaluation of stand-alone LLM anonymization approaches and quantum-enhanced hybrid architectures for the deidentification of Portuguese clinical notes. Specifically, we aimed to determine whether the proposed Dynex-QML framework could improve anonymization performance compared with conventional prompt-based LLM approaches, particularly regarding precision, false-positive (FP) reduction, and overall *F*_1_-score, while preserving high sensitivity and practical computational efficiency.

## Methods

### Database Construction and Preparation

#### Anonymization Corpus Development

A dedicated dataset was constructed at the Heart Institute (Instituto do Coração [InCor]), 1 of the 6 specialized institutes within the Hospital das Clínicas of the University of São Paulo Medical School (HC-FMUSP), the largest academic health care complex in Latin America. Although collected within a single health care system, the dataset reflects substantial heterogeneity due to the large and diverse patient population served by HC-FMUSP and the broad range of clinical documentation practices encountered in this setting. InCor, as a tertiary cardiovascular referral center, receives patients from multiple geographic regions of Brazil and from diverse socioeconomic backgrounds. Furthermore, the Brazilian population is characterized by a high degree of ethnic and cultural admixture, resulting from the historical contributions of European, African, Asian, and Indigenous populations. This diversity is reflected in clinical documentation through a wide variety of personal names, surnames, demographic characteristics, cultural references, and other potentially identifiable entities. Consequently, the dataset captures considerable variability in both patient-related information and physician documentation styles, providing a challenging and representative environment for evaluating anonymization methods. The dataset was specifically developed to validate the anonymization approaches evaluated in this study. This gold-standard corpus comprises 1000 outpatient clinical notes, originally written in Brazilian Portuguese, and reflects a broad range of sensitive information representative of real-world clinical documentation.

The clinical notes were extracted from the electronic patient record [42,43], covering encounters recorded between July 2020 and February 2024. Patients in the source dataset had a mean age of 62.5 (SD 18.37) years, with a balanced sex distribution, and the most frequent *ICD-10* (*International Statistical Classification of Diseases, Tenth Revision*) codes were mainly cardiovascular. The notes consisted primarily of patient anamneses from routine clinical care, and had a median length of 1168 (IQR 682.5-1645.5) characters. They included information on personal and family medical history, diagnoses, medications, laboratory and imaging test results, and clinical evolution. The source notes also contained a diverse range of sensitive and potentially identifying information commonly found in routine clinical documentation. All original patient information was replaced with pseudoinformation during the annotation process. In the following, we show an example of a clinical note from the dataset.

The study protocol was reviewed and approved in accordance with institutional and regulatory requirements by the relevant medical and scientific bodies and by the independent Institutional Review Board (CAPPesq – IRB Number 68, Brazilian National Research Ethics System), under registration number CAAE 89528325.8.0000.0068. All patient identifiers were replaced during the annotation process to ensure data protection and confidentiality.

To construct a reliable ground truth for evaluation, 5 annotators manually identified and replaced sensitive entities using standardized annotation tags. The annotation team consisted of doctor-level researchers with backgrounds in biomedical engineering and experience in natural language processing (NLP) applied to medical texts and clinical data.

Before the annotation process, all annotators received specific training on the 5 protected-entity categories, entity-boundary definitions, inclusion and exclusion criteria, and the use of standardized annotation tags. The training included the review of representative clinical-note examples and pilot annotation cases. Questions and ambiguous cases identified during this phase were discussed by the annotation team, and the annotation guidelines were refined before the final annotation process.

The corpus was divided into 5 independent subsets of 200 clinical notes, with each subset annotated by one annotator following predefined annotation guidelines specifying entity boundaries and classification criteria. To improve annotation consistency, the annotators participated in iterative guideline refinement discussions during corpus development. Ambiguous annotation cases and uncertain entity classifications were reviewed collaboratively and resolved through consensus discussion. The complete annotation guidelines are publicly available in the study’s GitHub repository.

To provide an additional assessment of annotation reliability, 100 clinical notes were randomly selected from the 500-note evaluation set. The same 100 original clinical notes were independently reannotated by 3 of the 5 annotators who had participated in the original corpus annotation. Each of the 3 annotators completed the annotation of all 100 notes independently using the same protected-entity definitions, annotation guidelines, and annotation procedures applied during the initial corpus construction.

The annotators were blinded to the original gold-standard annotations, the annotations produced by the other annotators, the pseudonymized texts subsequently generated from the original annotations, and all model outputs. The 3 reannotation sets were used exclusively for the interannotator agreement analysis and did not replace or modify the original gold-standard annotations used for model evaluation. The entity types and their characteristics are detailed in Table 1.

**Table 1.** Protected entity categories used for manual annotation of the gold-standard corpus of 1000 Portuguese outpatient clinical notes collected at a tertiary-care academic hospital in Brazil, including entity tags, semantic definitions, and representative examples used during anonymization benchmarking.

Entity tag	Description	Examples
<NAME>	Person’s name	Jessica, Miguel, Maria do Carmo
<DATE>	Dates in various formats	27/06/2022, Março, 13Fev, 2006
<ORGANIZATION>	Institution or company	InCor, UBS Perus/Lapa, ICESP
<LOCAL>	Geographic location	Piraporinha, São Paulo, Medina-MG
<ID>	Identifiers (patient ID, ZIP code, phone, and email)	12312312, XA323423, 04330‐010, 1198576‐3122, and maria@hotmail.com

#### Pseudonymization for Algorithm Validation

To avoid exposing real patient information during the algorithm validation process, a pseudonymized version of the texts was generated. In this version, each annotated entity was randomly replaced with a synthetic value drawn from appropriate reference lists:

1. <DATE**>**: Random dates between 1950 and 2024, generated in various formats commonly used in Brazilian clinical documentation (eg, 05/24/1990, 23 de maio de 2025, and 13-Fev-2006).
2. <NAME**>**: Brazilian names extracted from publicly available census lists, preserving the distribution of common first and family names.
3. <LOCAL**>**: Brazilian municipalities obtained from official geographic repositories maintained by the Instituto Brasileiro de Geografia e Estatística (IBGE).
4. <ORGANIZATION**>**: Names of Brazilian hospitals and health care facilities, sourced from open datasets of the Sistema Único de Saúde (SUS, Brazilian Unified Health System).

This specially constructed and manually annotated dataset provided a gold-standard benchmark for systematically testing and comparing anonymization algorithms while preserving the confidentiality of authentic clinical information.

### Anonymization Approaches

#### LLM-Based Anonymization Approach

To establish a performance baseline, we selected 2 publicly available LLMs from the Llama family: Llama-3.1-8B-instruct and Llama-3.3-70B-instruct. These models were chosen for their instruction-following capabilities and their suitability for local deployment, which addresses privacy concerns associated with transmitting clinical data to external services [28].

The methodology used a carefully crafted prompt designed to systematically direct the model to identify and replace all occurrences of each predefined category of sensitive information in the clinical notes. The prompt was developed iteratively, with refinements based on preliminary testing to optimize entity recognition across all categories. All evaluation runs used identical decoding settings across both LLM and Dynex-QML configurations to support a controlled comparison: temperature 0.1, top-p 0.95, maximum output length 4096 tokens, and a fixed random seed of 42. Both Llama models were served locally in 4-bit GPTQ quantization through the Hugging Face transformers and accelerate stack on the H100 SXM hardware described in Table 2, without any task-specific fine-tuning, in order to isolate the contribution of the optimization layer rather than the contribution of supervised adaptation. The text shown in Textbox 1 was supplied to the model as a single user-role message; no separate system prompt was used. The anonymization prompt used in the evaluation is shown in Textbox 2.

**Table 2.** Hardware configuration of the high-performance computing environment used for evaluating stand-alone LLMs^a^ and quantum-enhanced Dynex-QML anonymization models on Portuguese clinical notes (all evaluation runs reported in this paper were executed on the configuration shown).

Category	Details
CPU^b^	Intel Xeon Platinum, 16 cores / 128 threads (258/2064 GB RAM)
GPU^c^	1 × NVIDIA H100 SXM
GPU memory	80 GB (2507.0 GB/s, 478.1 GB/s)
GPU Performance	53.5 TFLOPS (Max CUDA: 12.6)
Motherboard or Series	The complete annotation guidelines are publicly Z13PN-D32 Series (PCIe 5.0, 16x, 52.7 GB/s)
Storage	Samsung MZQLB3T8HAJD-00007 (34,682 MB/s, 2827.3 GB)
Network	3233 Mbps upload/1298 Mbps download

a LLM: large language model.

b CPU: central processing unit.

c GPU: Graphics Processing Unit.

Textbox 1. **Representative pseudonymized Portuguese outpatient clinical note from the gold-standard corpus used to evaluate the anonymization models.**

SPA - retorno

<NAME>, 88 anos natural <LOCAL> / procedente de <LOCAL> / viúva / mora com filho / aposentada (costureira)

HD: achado incidental em Holter de BAV 2:1 e BAVT assintomática

Holter <DATE>: apenas BAV2M1 durante período noturno com FC 30

AP:

- HAS (dx: <DATE>)
- DRC
- IVP
- POT Histerectomia (mioma?)
- POT Colecistectomia
- G1P1A0C0

MUC:

- HCTZ 25 mg 1-0-0
- Anlodipino 5 mg 1-0-0
- Captopril 25 mg 1-0-0
- Omeprazol 20 mg 1-0-0

AF:

- mãe: já teve MP

HMA:

- achado incidental em Holter de BAV 2:1 e BAVT externo - sem data - sem traçado
- atividade física: não
- dispneia para esforço: nega
- síncope: nega
- tontura: nega

EXAME:

- neuro: taquilálica
- acv: PA 220 × 100/ FC 62 - regular com batimento precoce / B1 normo, sopro sist FMi 2+/6+ regurgitativo, B2 normo / sem congestão

EXAMES:

- Holter <DATE>: 76,407 batimentos (mín/med/max - 30/58/132) / ectópicos ventriculars (1136 - isoladas 50, bigeminismo 62 - pares 47 - TVNS 5 (maior 04 batimentos) / ectópicos supra (970 - TANS maior 94 batimentos e 50s / BAV2M1 durante o sono / não relatou sintomas / episódios de ritmo de escape juncional

CD:

- achados compatíveis com doença de condução, porém sem BAV avançado ou pausas - encaminho para Marca-Passo para seguimento

<NAME> r1 cardio + Dr <NAME>

Textbox 2. **Anonymization prompt used for prompt-based anonymization of Portuguese clinical notes. The prompt instructed the models to identify and replace protected health information entities using predefined anonymization tags.**

Anonymize the following medical text by replacing all Protected Health Information (PHI) with appropriate tags.

Use the following tags:

- <NAME> for any person names (patients, doctors, etc)
- <ORGANIZATION> for organization names (hospitals, clinics, etc)
- <LOCAL> for location information
- <ID> for any identification numbers
- <DATE> for any dates

Return only the anonymized text, with no additional explanations or comments.

Here is the text to anonymize:

#### Quantum-Enhanced Hybrid Anonymization Approach (Dynex-QML)

A quantum machine learning architecture was implemented to evaluate the efficacy of quantum-enhanced text processing for medical anonymization. This approach, designated Dynex-QML, used the same 2 LLMs as foundational components (Llama-3.1-8B-instruct and Llama-3.3-70B-instruct) but integrated neuromorphic quantum optimization algorithms to enhance the anonymization process. A note on terminology is in order before the technical description that follows. Throughout this paper, we use the term quantum-enhanced consistently in preference to quantum-inspired, since the Dynex platform (developed by Dynex Quantum Technologies AG) is a neuromorphic quantum computing platform that emulates the dynamics of quantum annealing through systems of ordinary differential equations integrated on Graphics Processing Unit (GPU) hardware, rather than a heuristic loosely inspired by quantum mechanics. The platform is also not a gate-model quantum computer with physical qubits in superposition; the qubits in our pipeline are algorithmic qubits, that is, mathematical representations of quantum states whose evolution is governed by memristive ordinary differential equation (ODE) dynamics solved within GPU video memory. We therefore make no claim of quantum speedup against classical optimization, and the gains reported in this work should be interpreted as gains from imposing a global QUBO-formulated optimization layer on top of standard LLM token selection, made tractable by the Dynex neuromorphic platform. The following subsections provide a detailed explanation of the quantum-enhanced methodology.

### Fundamentals of Quantum Annealing and QUBO Formulation

Quantum annealing is an optimization technique that exploits quantum mechanical phenomena to find the global minimum of an objective function [44]. The method is particularly effective for solving combinatorial optimization problems that can be formulated as finding the ground state (lowest energy configuration) of a quantum system.

The mathematical foundation of quantum annealing rests on the Ising model [45,46], which describes a system of interacting binary spins. For a system with N binary variables $s_{i}\in \{-1,+1\}$, the energy function (Hamiltonian) is expressed as:


(1) $$H\left(s\right)=\;\sum_{i}h_{i}s_{i}+\;\sum_{i<j}J_{ij}s_{i}s_{j}$$

where $h_{i}$ represents the local field acting on spin i, and $J_{ij}$ represents the interaction strength between spins i and j. The goal is to find the spin configuration that minimizes this energy function.

For computational convenience, the Ising model can be reformulated as a QUBO problem by transforming the binary variables from {-1,+1} to {0,1} domain using the substitution$s_{i}=2x_{i}-1$. The resulting QUBO formulation is:


(2) $$E\left(x\right)=\;\sum_{i}Q_{ii}x_{i}+\;\sum_{i<j}Q_{ij}x_{i}x_{j}$$

where $x_{i}\in \{0,1\}$ and Q are real-valued matrices encoding both the linear and quadratic terms of the objective function. This formulation is particularly suitable for representing constraint satisfaction problems and optimization tasks that arise in NLP [35,47].

### Transformer Architecture and Attention Mechanism

Modern LLMs are built on the transformer architecture, which relies fundamentally on the attention mechanism to process sequential data [48]. The multihead attention mechanism computes weighted representations of input tokens by learning which parts of the input sequence are most relevant for predicting each output token.

For a given input sequence, the attention mechanism computes 3 matrices: Query (Q), Key (K), and Value (V), derived from the input embeddings through learned linear transformations. The attention weights are computed as:


(3) $$Attention\left(Q,\;K,\;V\right)=\;softmax\;\left(\frac{QK^{t}}{\sqrt{d_{k}}}\right)\;V$$

where $d_{k}$ is the dimensionality of the key vectors. The softmax operation normalizes the attention scores to create a probability distribution over the input tokens.

In a typical transformer-based LLM, multiple layers of multihead attention are stacked, with each layer refining the representations. The final layer produces output logits that are passed through a softmax function to generate probability distributions over the vocabulary for each token position. In text generation tasks, including anonymization, these probabilities guide the selection of output tokens.

### Dynex Neuromorphic Quantum Computing Platform

The Dynex platform implements neuromorphic quantum computing through an architecture that emulates quantum behavior using memristor-based circuits [37]. Unlike gate-based quantum computers that operate on physical qubits in quantum superposition, Dynex uses algorithmic qubits, that is, mathematical representations of quantum states that are simulated within GPU video random access memory (VRAM).

The core innovation of the Dynex platform lies in its ability to construct problem-specific quantum circuits composed of linear and quadratic quantum gates. These gates are not physical quantum components but rather mathematical models of memristive elements whose behavior is governed by systems of ODEs. The platform solves these ODEs through numerical integration to simulate the quantum annealing process.

For linear quantum gates representing individual qubits, the voltage dynamics are described by:


(4) $$v_{n}=\;\sum_{m}QG_{m}W_{m}p_{i}$$

where $v_{n}$ is the qubit voltage (representing the qubit state), $QG_{m}$ is the quantum gate state computed as $QG_{m}=(1.0-p_{i}v_{i})/2.0$, $W_{m}$ is the quantum gate weight (encoding the QUBO coefficients), and $p_{i}$ is the polarity indicator (+1 for i§amp;gt;0, -1 for i≤0).

For quadratic quantum gates representing interactions between qubits, more complex dynamics govern the system evolution, incorporating both gradient terms and rigidity constraints that ensure the quantum circuit converges to a stable solution. The platform automatically optimizes the annealing parameters (α,β,γ,δ,ϵ,ε,ζ) for each problem class to maximize solution quality.

The use of memristor-based simulation allows the Dynex platform to leverage the massive parallel processing capabilities of modern GPUs while maintaining the mathematical properties of quantum annealing. This approach provides computational efficiency that scales with the number of GPU cores available, enabling practical deployment of quantum-inspired algorithms on existing hardware infrastructure.

### Quantum-Classical Integration via Dynex-QML

#### Overview

The Dynex-QML architecture integrates the classical transformer-based LLM with quantum optimization through a designed interface that we term “Quantum Bridges.” These bridges enable integration between popular deep learning frameworks (PyTorch and TensorFlow) and the Dynex quantum computing platform.

The integration process consists of the following steps:

#### Step 1: Extraction of Attention Layer

Rather than allowing the LLM to generate output tokens through standard sequential sampling, we intercept the model’s computation at the final attention layer. Specifically, we extract the attention weight matrices before they are converted to output probabilities. For a sequence of length L with vocabulary size V, this produces a tensor of shape (L,V) representing the unnormalized scores for each token at each position.

#### Step 2: Reformulation as Global Constraint Satisfaction Problem

Traditional LLM text generation proceeds left-to-right, selecting each token sequentially based on previously generated tokens. This local decision-making can lead to suboptimal global choices, particularly in constrained generation tasks such as anonymization where maintaining semantic coherence while replacing entities is crucial.

We reformulate the anonymization task as a Global Constraint Satisfaction Problem (GCSP) where all token decisions across the entire output sequence are optimized simultaneously. The objective is to find a configuration of output tokens that maximizes overall coherence while satisfying the anonymization constraints (correct entity type replacement, preservation of nonsensitive text, and maintenance of grammatical structure).

#### Step 3: Transformation to QUBO Formulation

The GCSP is encoded as a QUBO problem where binary variables represent token selection decisions. For each position i in the output sequence and each possible token t from the vocabulary, we introduce a binary variable $x_{i,t}\in \{0,1\}$ indicating whether token t is selected at position i.

The QUBO objective function is constructed to incorporate multiple terms:


(5) $$E\left(x\right)=E_{LLM}\left(x\right)+\lambda_{1}E_{constraint}\left(x\right)+\lambda_{2}E_{coherence}\left(x\right)$$

where $E_{LLM}(x)$ encodes the negative log-probabilities from the LLM’s attention layer, favoring tokens that the base model considers likely; $E_{constraint}(x)$ encodes hard constraints (eg, exactly one token selected per position and entity type consistency); $E_{coherence}(x)$ encodes soft constraints promoting semantic coherence across the entire output; and $\lambda_{1}$ and $\lambda_{2}$ are hyperparameters balancing the different objectives. In the evaluation reported in this paper, the constraint-penalty weight was set to a value sufficiently large relative to the LLM energy scale to enforce hard one-token-per-position assignment and tag-schema consistency (specifically, lambda_1 was chosen as approximately 10 times the maximum absolute attention-derived coupling magnitude observed on the development split), while the coherence weight lambda_2 was selected on the development split of 500 notes via grid search over the set {0.1, 0.25, 0.5, 1.0, 2.0} to maximize macro*–F*_1_-score, with the value 0.5 ultimately retained for the test-set evaluation. The Dynex annealing scheduler parameters α, β, γ, δ, ε, varepsilon, and ζ were left at the platform’s automatic per-problem-class defaults, which the Dynex platform selects by analyzing the structure of the submitted QUBO matrix; we did not override these values.

The attention weights from the LLM directly influence the Q matrix coefficients in the QUBO formulation. High attention weights between positions translate to stronger coupling terms Q_ij_ in the QUBO matrix, ensuring that the quantum optimization respects the semantic relationships learned by the LLM.

#### Step 4: Quantum Annealing via Dynex Platform

The QUBO problem is submitted to the Dynex platform, which constructs the corresponding neuromorphic quantum circuit. The circuit is composed of linear quantum gates for each binary variable $x_{i,t}$, encoding the unary terms from $E_{LLM}$ and $E_{constraint}$; and quadratic quantum gates for pairs of variables, encoding the pairwise interaction terms from $E_{coherence}$ and the coupling derived from attention weights.

The platform then performs quantum annealing by numerically integrating the ODEs governing the memristive quantum gates. The annealing process gradually evolves the system from a superposition of all possible configurations toward the ground state (minimum energy configuration), which corresponds to the optimal anonymization.

#### Step 5: Solution Extraction and Decoding

Upon convergence, the final state of the quantum circuit provides the binary values for all variables $x_{i,t}$. These are decoded back to the output token sequence, producing the anonymized text. The quantum optimization ensures that this sequence simultaneously satisfies the anonymization requirements while maintaining maximal coherence with the original clinical meaning.

The Quantum Bridge implementation handles the technical details of converting PyTorch or TensorFlow tensors into QUBO format, submitting the problem to the Dynex API, and translating the quantum solution back into the framework’s native tensor format. This abstraction allows researchers to incorporate quantum optimization into existing LLM pipelines with minimal code modifications.

Through this Dynex-QML formulation, the quantum machine learning model generates coherent anonymized outputs based on quantum-optimized attention states while maintaining semantic integrity throughout the anonymization process. The global optimization approach addresses the precision issues observed in standard prompt-based LLM anonymization by avoiding the local decision-making that can lead to over-identification of entities.

### Evaluation Metrics

To assess the effectiveness and efficiency of the anonymization methods, we evaluated both quality-related and computational performance metrics on 500 of the 1000 clinical texts in the dataset, selected at random to constitute the test set.

### Quality Metrics

The quality of anonymization for each method was quantitatively determined based on three main criteria:

1. True positives (TP): Terms that should have been anonymized and were correctly anonymized with the appropriate entity tag.
2. False positives (FP): Items that should not have been anonymized but were erroneously marked for anonymization.
3. False negatives (FN): Terms that should have been anonymized but were missed by the algorithm.

These counts served as the basis for calculating the primary quality metrics for each entity type considered (<NAME>, <DATE>, <LOCAL>, <ORGANIZATION>, and <ID>):


(6) $$Precision=\;\frac{TP}{TP+FP}$$

(7) $$Recall\;\left(sensitivity\right)=\;\frac{TP}{TP+FN}$$

(8) $$F_{1}-score=2\;\times \;\frac{Precision\;\times \;Recall}{Precision+\;Recall}$$

The macro*–F*_1_-score was calculated as the unweighted mean of *F*_1_-scores across all entity types to provide an overall performance measure. To estimate statistical uncertainty, 95% CIs for precision, sensitivity (recall), and *F*_1_-score were computed using bootstrap resampling based on aggregate multinomial TP, FP, and FN counts for each model and entity category. For each bootstrap iteration, TP, FP, and FN counts were resampled from the observed aggregate count distribution, and the corresponding metrics were recomputed. We used 10,000 bootstrap iterations. Macro-level CIs were calculated by averaging the bootstrapped entity-level metrics across the 5 entity categories.

Pairwise model comparisons were performed using empirical 2-sided bootstrap tests on the difference in *F*_1_-score. Prespecified within-size comparisons were Dynex-QML (Llama 8B) versus stand-alone Llama 3.1 8B and Dynex-QML (Llama 70B) versus stand-alone Llama 3.3 70B. For each comparison, the empirical *P* value was calculated as twice the smaller tail probability of the bootstrap distribution of the *F*_1_-score difference. Statistical significance was defined as *P*<.05.

For computational performance, uncertainty in mean internal processing time (TI) and mean total elapsed time (TT) was estimated using note-level bootstrap resampling of the 500 observed per-note timing measurements for each model. Pairwise time comparisons used paired bootstrap resampling by clinical note ID, preserving the matched structure of the timing data across models. Empirical 2-sided *P* values were calculated from the bootstrap distribution of paired mean time differences, with statistical significance defined as *P*<.05.

### Interannotator Agreement Analysis

Interannotator agreement was evaluated using the 3 independent annotation sets produced by the 3 annotators for the same 100 randomly selected clinical notes. Pairwise comparisons were performed for the 3 possible annotator pairs.

Agreement was assessed at the entity level for NAME, DATE, ID, LOCAL, and ORGANIZATION. An entity was considered concordant only when both annotators identified the same complete text span and assigned the same protected-entity category.

Entity-level *F*_1_-scores were calculated separately for each protected-entity category and annotator pair. For each category, the mean pairwise *F*_1_-score was calculated as the arithmetic mean of the 3 pairwise comparisons. The macro*–F*_1_-score for each annotator pair was calculated as the unweighted mean of the 5 entity-level *F*_1_-scores. The overall mean pairwise macro*–F*_1_-score was calculated as the arithmetic mean of the 3 pairwise macro*–F*_1_-scores. Percentile-based 95% CIs were estimated using 1000 note-level bootstrap samples with replacement.

### Ethical Considerations

This study was conducted in accordance with the Institutional Review Board (CAPPesq – IRB Number 68, Brazilian National Research Ethics System), under registration number CAAE 89528325.8.0000.0068. The clinical notes were retrospectively obtained from routine clinical care. Original notes were accessed only by authorized researchers within a secure institutional environment for the manual annotation procedure. Before model development and evaluation, all annotated identifiers were replaced with synthetic values to generate pseudonymized clinical texts. The ethics committee waived the requirement for individual informed consent because of the retrospective study design and the data-protection measures adopted. All data-handling procedures complied with the Brazilian General Data Protection Law, LGPD.

### Computational Performance Metrics

The Heart Institute research team executed all anonymization algorithms on remote high-performance servers to ensure reproducible computational servers provided by Dynex within a Sandbox environment. For each model evaluation, 2 complementary time metrics were recorded to characterize computation performance:

- TI: The inference time reported directly by the execution platform reflecting the model’s internal computation and excluding any network communication overhead.
- TT: The complete time required to process each request, including both model inference and all network-related latency associated with transmitting data to and from the remote servers.

Both processing time metrics were recorded for each text and aggregated to provide per-text averages (mean and SD) and totals for the complete test set of 500 clinical notes. This dual-metric approach enables a clearer distinction between intrinsic model efficiency and external latency effects introduced by remote execution. TI reflects the intrinsic computational efficiency of the anonymization model itself, whereas TT better approximates the latency experienced in real-world deployment scenarios where remote execution and network communication are required.

The evaluation framework was assessed using the following standard classification metrics: TP, FP, FN, precision, recall (sensitivity), and *F*_1_-score, along with processing time (TI and TT) to capture computational efficiency.

## Results

### Computing Environment

Table 2 summarizes the computing environment used for all model evaluations, including hardware specifications and software configurations that supported the execution of both LLM-based and quantum-enhanced models.

### Interannotator Agreement

The same 100 randomly selected clinical notes were independently reannotated by 3 annotators. Pairwise entity-level agreement results and the numbers of entities identified by each annotator are presented in Table 3. The overall mean pairwise macro*–F*_1_-score across the 5 protected-entity categories was 0.786 (SD 0.038; 95% CI 0.700‐0.869). Pairwise macro*–F*_1_-scores ranged from 0.744 to 0.816.

**Table 3.** Pairwise entity-level interannotator agreement among 3 annotators who independently reannotated the same 100 randomly selected Portuguese clinical notes^a^.

Entity	Entity count (A1/A2/A3)	*F*_1_-score (A1 vs A2)	*F*_1_-score (A1 vs A3)	*F*_1_-score (A2 vs A3)	Mean pairwise *F*_1_-score (SD)
NAME	59/58/57	0.974	0.948	0.974	0.966 (0.015)
DATE	318/323/307	0.958	0.957	0.927	0.947 (0.018)
ID	6/5/1	0.545	0.286	0.333	0.388 (0.138)
LOCAL	30/36/28	0.727	0.931	0.688	0.782 (0.131)
ORGANIZATION	66/71/62	0.876	0.875	0.797	0.849 (0.045)
Macro*–F*_1_-score	—^b^	0.816	0.799	0.744	0.786 (0.038)

a A1, A2, and A3 represent anonymized annotator identifiers. Entity counts indicate the number of entities identified by each annotator. An entity was considered concordant only when the same complete text span and protected-entity category were assigned by both annotators. Mean pairwise *F*_1_-score represents the arithmetic mean of the 3 pairwise comparisons. Macro*–F*_1_-score represents the unweighted mean across NAME, DATE, ID, LOCAL, and ORGANIZATION.

b The entity count is not applicable to the macro*–F*_₁_-score row.

Agreement was highest for NAME, with a mean pairwise *F*_1_-score of 0.966 (SD 0.015; 95% CI 0.929‐0.990), followed by DATE at 0.947 (SD 0.018; 95% CI 0.928‐0.963), ORGANIZATION at 0.849 (SD 0.045; 95% CI 0.793‐0.897), and LOCAL at 0.782 (SD 0.131; 95% CI 0.649‐0.895). ID showed the lowest mean pairwise *F*_1_-score at 0.388 (SD 0.138; 95% CI 0.000‐0.778).

### Anonymization Quality

The full set of anonymization quality metrics is presented in Table 4. Performance varied substantially across entity categories and model architectures. Dynex-QML (Llama 70B) achieved the highest macro*–F*_1_-score (0.855, 95% CI 0.823‐0.880), followed by Dynex-QML (Llama 8B; 0.733, 95% CI 0.709‐0.756), stand-alone Llama 3.3 70B (0.726, 95% CI 0.704‐0.747), and stand-alone Llama 3.1 8B (0.602, 95% CI 0.588‐0.615). At the macro level, Dynex-QML (Llama 70B) improved *F*_1_-score by 0.128 compared with stand-alone Llama 3.3 70B (95% CI 0.091‐0.163; empirical 2-sided bootstrap *P*<.001). Dynex-QML (Llama 8B) improved macro*–F*_1_-score by 0.131 compared with stand-alone Llama 3.1 8B (95% CI 0.104‐0.158; *P*<.001).

**Table 4.** Entity-level anonymization performance of 4 evaluated models on 500 held-out Portuguese outpatient clinical notes from a tertiary-care academic hospital in Brazil^a^.

Entity and model	TP^b^	FP^c^	FN^d^	Precision	Sensitivity	*F*_1_-score	Count
NAME							
Dynex-QML (70B)	329	10	17	0.971	0.951	0.961	346
Dynex-QML (8B)	335	207	11	0.618	0.968	0.755	346
Llama 3.3 70B	335	202	11	0.624	0.968	0.759	346
Llama 3.1 8B	268	208	78	0.563	0.775	0.652	346
DATE							
Dynex-QML (70B)	1624	68	16	0.960	0.990	0.975	1640
Dynex-QML (8B)	1604	132	36	0.924	0.978	0.950	1640
Llama 3.3 70B	1606	141	34	0.919	0.979	0.948	1640
Llama 3.1 8B	1555	450	85	0.776	0.948	0.853	1640
ID							
Dynex-QML (70B)	20	32	1	0.385	0.952	0.548	21
Dynex-QML (8B)	17	81	4	0.173	0.810	0.286	21
Llama 3.3 70B	18	107	3	0.144	0.857	0.247	21
Llama 3.1 8B	19	645	2	0.029	0.905	0.055	21
LOCAL							
Dynex-QML (70B)	273	24	5	0.919	0.982	0.950	278
Dynex-QML (8B)	267	32	11	0.893	0.960	0.925	278
Llama 3.3 70B	267	39	11	0.873	0.960	0.914	278
Llama 3.1 8B	262	189	16	0.581	0.942	0.719	278
ORGANIZATION							
Dynex-QML (70B)	335	122	7	0.733	0.980	0.839	342
Dynex-QML (8B)	334	215	8	0.608	0.977	0.750	342
Llama 3.3 70B	335	203	7	0.623	0.980	0.761	342
Llama 3.1 8B	286	156	56	0.647	0.836	0.730	342

a Results are reported separately for NAME, DATE, ID, LOCAL, and ORGANIZATION entities using true positives (TP), false positives (FP), false negatives (FN), precision, sensitivity, and *F*_1_-score. Values are point estimates. The bootstrap 95% CIs and statistical comparisons for *F*_1_-score are shown in Figure 1.

b TP: true positive.

c FP: false positive.

d FN: false negative.

**Figure 1.**
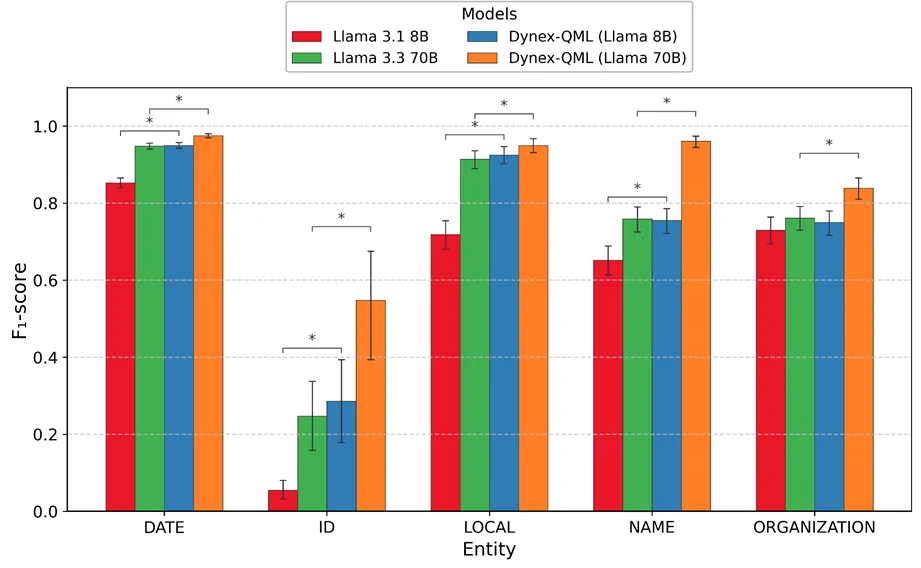
Comparison of entity-level *F*_1_-scores across 4 anonymization models, Llama 3.1 8B, Llama 3.3 70B, Dynex-QML 8B, and Dynex-QML 70B, evaluated on 500 held-out Portuguese outpatient clinical notes from a tertiary-care academic hospital in Brazil. The evaluated protected entity categories included DATE, ID, LOCAL, NAME, and ORGANIZATION. Error bars indicate aggregate multinomial bootstrap 95% CIs. Brackets indicate pairwise comparisons. All starred comparisons had *P*<.001, except Llama 3.3 70B vs Dynex-QML 70B for LOCAL (*P*=.03).

**Figure 2.**
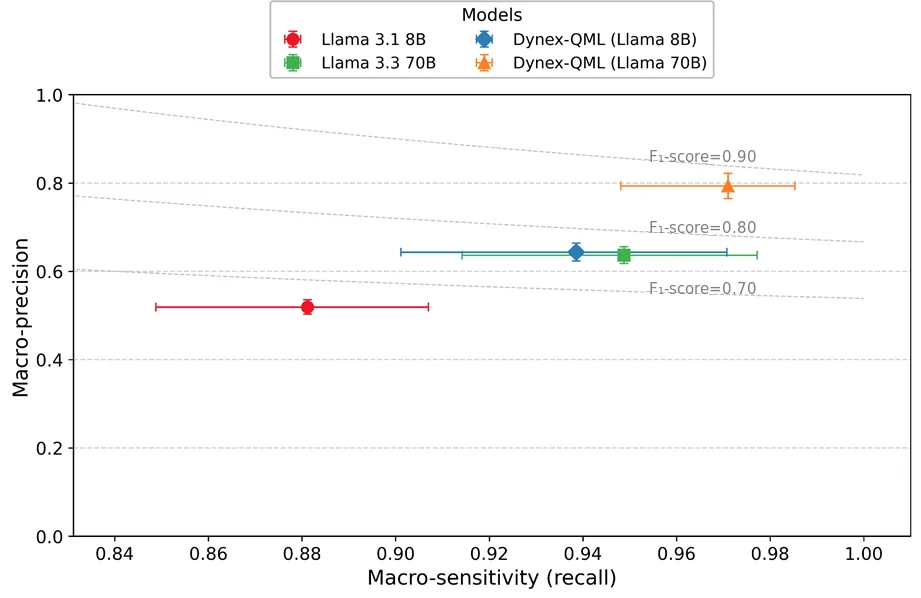
Macro-sensitivity (recall) and macro-precision trade-off of 4 anonymization models evaluated on 500 held-out Portuguese outpatient clinical notes from a tertiary-care academic hospital in Brazil. Each point represents one anonymization model (Llama 3.1 8B, Llama 3.3 70B, Dynex-QML 8B, and Dynex-QML 70B), with horizontal and vertical error bars indicating aggregate bootstrap 95% CIs for macro-sensitivity and macro-precision, respectively. Dashed diagonal lines represent iso*–F*_1_-score contours. Points located toward the upper-right region indicate a more favorable balance between privacy protection, represented by sensitivity, and preservation of clinical utility, represented by precision.

Figure 1 displays the entity-level *F*_1_-score comparison across all protected entity categories for the 4 evaluated models. To further characterize model behavior beyond *F*_1_-score, Figure 2 shows the macro-sensitivity and macro-precision trade-off across architectures. Dynex-QML (Llama 70B) achieved the most favorable overall balance, combining higher macro-precision with preserved macro-sensitivity compared with the other evaluated models.

**Figure 3.**
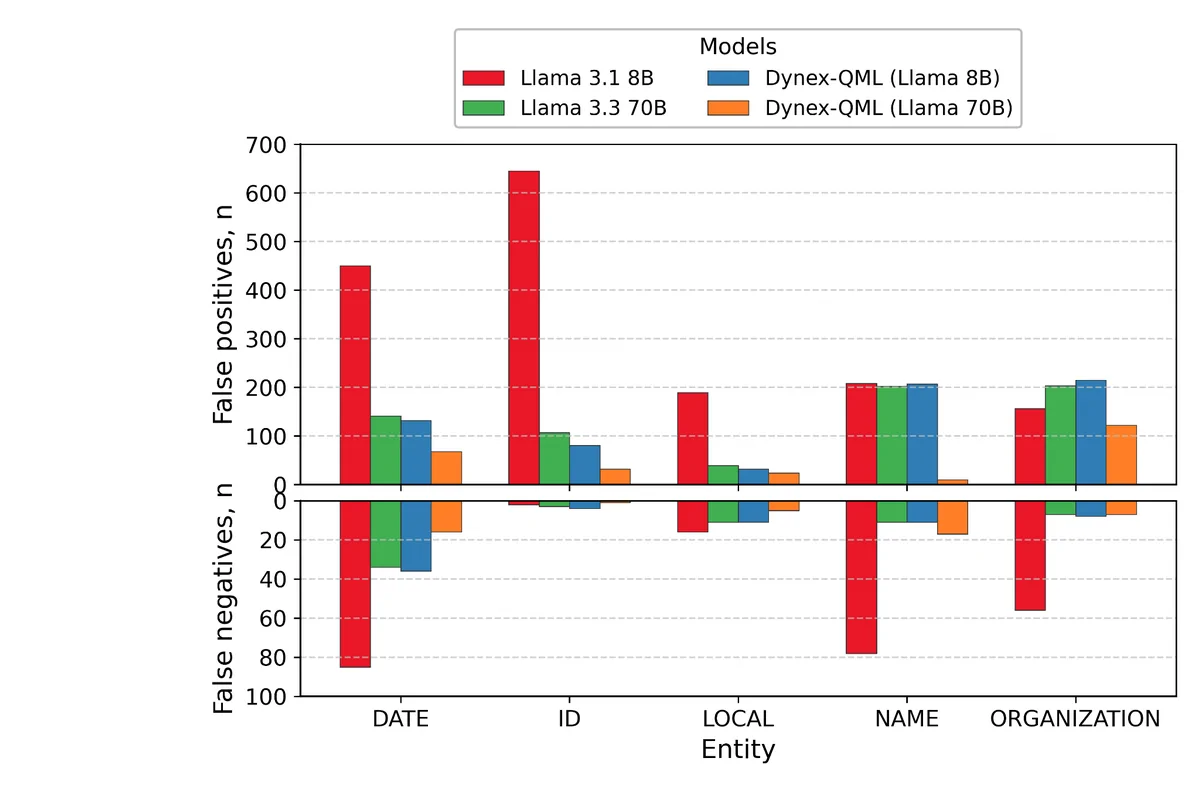
Entity-level false-positive and false-negative counts of 4 anonymization models evaluated on 500 held-out Portuguese outpatient clinical notes from a tertiary-care academic hospital in Brazil. Results are shown separately for DATE, ID, LOCAL, NAME, and ORGANIZATION entities. False positives represent overanonymization and potential loss of clinical information utility, whereas false negatives represent missed protected health information entities and potential privacy risk.

Figure 3 provides an entity-level error analysis by separating FP and FN counts across protected entity categories. In the context of clinical text anonymization, FNs are particularly important because they represent missed protected health information and therefore potential residual privacy risk, whereas FPs mainly represent overanonymization and potential loss of clinical information utility. Across entity categories, Dynex-QML (Llama 70B) reduced FP counts substantially compared with stand-alone Llama 3.3 70B, especially for NAME, ID, LOCAL, and ORGANIZATION entities. Importantly, this reduction in overanonymization was not accompanied by an increase in FN. The model maintained similarly low FN counts for DATE, NAME, LOCAL, and ORGANIZATION entities, and only one FN for ID entities. These results suggest that the main advantage of Dynex-QML (Llama 70B) was not improved precision alone, but a more favorable privacy-utility trade-off, combining high sensitivity with fewer unnecessary anonymization errors.

### Computational Performance

#### Overview

Computational performance is summarized in Table 5. For TT, stand-alone Llama 3.1 8B had the shortest mean processing time (5.22, SD 2.12; 95% CI 5.04‐5.41 seconds per note), followed by Dynex-QML (Llama 70B; 7.97, SD 2.87; 95% CI 7.72‐8.21 seconds per note), stand-alone Llama 3.3 70B (8.52, SD 3.32; 95% CI 8.23‐8.81 seconds per note), and Dynex-QML (Llama 8B; 8.59, SD 3.62; 95% CI 8.27‐8.90 seconds per note). In paired note-level bootstrap comparisons, Dynex-QML (Llama 70B) was significantly faster than stand-alone Llama 3.3 70B for TT, with a mean difference of −0.55 (SD 2.36; 95% CI −0.75 to −0.34 seconds per note; *P*<.001). The same pattern was observed for TI, with Dynex-QML (Llama 70B) being faster than stand-alone Llama 3.3 70B by −0.32 (SD 2.28; 95% CI −0.52 to −0.12 seconds per note; *P*=.002).

From an operational perspective, TT may represent a more clinically relevant metric because it captures effective end-to-end latency, including network communication and remote execution overhead commonly present in distributed health care infrastructures.

The computational trade-off differed for the 8B models. Dynex-QML (Llama 8B) was significantly slower than stand-alone Llama 3.1 8B for TT (+3.37, SD 2.77; 95% CI 3.13‐3.61 seconds per note; *P*<.001) and TI (+3.52, SD 2.63; 95% CI 3.29‐3.75 seconds per note; *P*<.001). Dynex-QML (Llama 70B) was also faster than Dynex-QML (Llama 8B) for TT (−0.62, SD 2.49; 95% CI −0.84 to −0.40 seconds per note; *P*<.001). These results indicate that the time-efficiency benefit was specific to the 70B Dynex-QML configuration and should not be generalized to all hybrid QUBO-optimized configurations.

**Figure 4.**
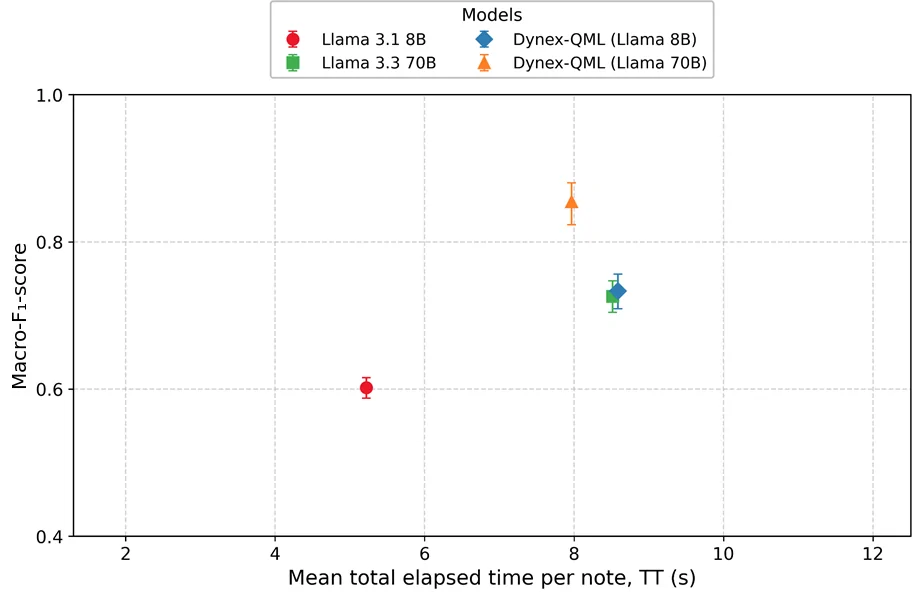
Efficiency-quality trade-off of the 4 anonymization models evaluated in this study: Llama 3.1 8B, Llama 3.3 70B, Dynex-QML 8B, and Dynex-QML 70B. The evaluation used 500 held-out Portuguese outpatient clinical notes from a single tertiary-care institution. The *x*-axis shows mean total elapsed time per note (TT, seconds), and the *y*-axis shows macro*–F*_1_-score across the 5 protected entity categories. Vertical error bars show bootstrap 95% CIs for macro*–F*_1_-score.

Figure 4 provides an overall assessment of the efficiency-quality trade-off across models using a scatter plot of macro*–F*_1_-score versus mean total elapsed time per note.

**Table 5.** Computational performance of stand-alone LLM^a^ and quantum-enhanced Dynex-QML anonymization models evaluated on 500 held-out Portuguese outpatient clinical notes^b^.

Model	TI^c^ (seconds), mean (SD; 95% CI)	TT^d^ (seconds), mean (SD; 95% CI)	Total time (seconds; 500 texts)
Llama 3.1 8B	3.92 (2.01; 3.75‐4.11)	5.22 (2.12; 5.04‐5.41)	2610.25
Llama 3.3 70B	7.22 (3.29; 6.94‐7.50)	8.52 (3.32; 8.23‐8.81)	4257.86
Dynex-QML (8B)	7.44 (3.58; 7.13‐7.75)	8.59 (3.62; 8.27‐8.90)	4293.27
Dynex-QML (70B)	6.90 (2.86; 6.65‐7.15)	7.97 (2.87; 7.72‐8.21)	3983.50

a LLM: large language model.

b Values are reported as mean seconds per note, with note-level bootstrap 95% CIs. Total time indicates the observed total elapsed TT for the 500-note test set.

c TI: internal processing time excluding network communication overhead.

d TT: total elapsed time, including remote execution, and network latency. Lower TT values may better reflect practical deployment performance in distributed health care infrastructures.

#### Representative Error Analysis

To support qualitative interpretation of the aggregate error counts reported in Table 4, Figure 3, and Table 6 presents representative pseudonymized error examples from the held-out test set. These examples are illustrative and are not intended to quantify error frequency. They show that errors occurred through different mechanisms, including overanonymization of non-PHI text, misclassification of entity categories, and missed protected entities.

**Table 6.** Representative pseudonymized error examples from the held-out test set of 500 Portuguese outpatient clinical notes^a^.

Model	Error type	Example	Gold standard	Model output	Comments
Llama 3.3 70B	False positive, NAME	“Profissão: gerente de oficina. Religião: protestante. Escolaridade: segundo grau completo...”	Sociodemographic terms should remain unchanged.	“Profissão: <NAME>. Religião: <NAME>. Escolaridade: <NAME>.”	The stand-alone model overanonymized non-PHI^b^ sociodemographic information as person names.
Llama 3.3 70B	False positive, DATE	“Queimação no peito há 10 anos... queimação no tórax há 10 anos.”	Clinical durations should remain unchanged.	“Queimação no peito há <DATE>... queimação no tórax há <DATE>.”	The stand-alone model incorrectly treated symptom duration as a calendar date.
Llama 3.3 70B	False negative, NAME	“ID: Sálvio, 15...”	“ID: <NAME>, 15...”	“ID: <ID>, 15...”	The patient name was detected as sensitive text but assigned to the wrong entity class.
Llama 3.3 70B	False negative, ID	“1° atendimento - 30-09-1987 Lubomira 34636-SP.”	“1° atendimento - <DATE> <NAME> <ID>.”	“1° atendimento - <DATE> <NAME> <LOCAL>.”	The identifier was detected but misclassified as a location, producing an ID false negative.
Dynex-QML 70B	False positive, LOCAL	“...procurou serviço médico no São José de Piranhas...”	“...serviço médico no <LOCAL>...”	“...serviço médico no <ORGANIZATION>...”	Dynex-QML 70B misclassified a municipality as an organization.
Dynex-QML 70B	False positive, ORGANIZATION	“pedido de consulta da PNEUMOLOGIA - A2MP - AMBULATORIO...”	This service-header phrase was not tagged in the gold standard.	“pedido de consulta da <ORGANIZATION> - <ORGANIZATION> - <ORGANIZATION>...”	Dynex-QML 70B produced repeated organization tags in a header that was not annotated as PHI.
Dynex-QML 70B	False negative, NAME	“Gladyr, 96 anos, natural e procedente de Ibitiara...”	“<NAME>, 96 anos, natural e procedente de <LOCAL>...”	“Gladyr, 96 anos, natural e procedente de <LOCAL>...”	Dynex-QML 70B left the patient name visible.
Dynex-QML 70B	False negative, ORGANIZATION	“Encaminhado do PS valore day hospital.”	“Encaminhado do PS <ORGANIZATION>.”	“Encaminhado do PS valore day hospital.”	Dynex-QML 70B missed the referring hospital name.

a The table compares selected gold-standard annotations with model outputs for stand-alone Llama 3.3 70B and Dynex-QML 70B. Error types were assigned by the authors after manual review. The examples are intended to illustrate qualitative error modes, not to quantify their frequency.

b PHI: Protected Health Information.

For stand-alone Llama 3.3 70B, common qualitative errors included FP anonymization of sociodemographic or clinical expressions, such as occupation, religion, schooling, and symptom duration, as well as wrong entity assignment when sensitive spans were detected but mapped to an incorrect category. For Dynex-QML 70B, the selected examples show residual errors despite the overall reduction in FP, including organization-location ambiguity, repeated organization tagging in service-header text, missed patient names, and missed organization mentions.

These examples are consistent with the quantitative results in Table 4 and Figure 3: Dynex-QML 70B reduced several overanonymization patterns compared with stand-alone Llama 3.3 70B, but it did not eliminate all clinically relevant errors. Therefore, the qualitative analysis supports the interpretation that the main benefit of Dynex-QML 70B was an improved privacy-utility trade-off rather than error-free anonymization.

## Discussion

### Principal Findings

This study presents a comparative assessment of anonymization performance using both conventional LLMs and a quantum-enhanced hybrid architecture (Dynex-QML). Based on the primary macro*–F*_1_-score metric, Dynex-QML-70B achieved the best overall performance (0.855, 95% CI 0.823‐0.880), followed by Dynex-QML-8B, Llama-3.3-70B, and Llama-3.1-8B. The observed macro*–F*_1_-score improvement of Dynex-QML (Llama 70B) over stand-alone Llama-3.3-70B was supported by bootstrap analysis (mean difference +0.128, 95% CI 0.091‐0.163; *P*<.001). Entity-level analyses showed statistically significant improvements for most categories, with the strongest gains for ID and NAME entities. Computationally, Dynex-QML (Llama 70B) also showed a statistically significant reduction in total elapsed time compared with stand-alone Llama 3.3 70B (mean paired difference −0.55, SD 2.36; 95% CI −0.75 to −0.34 seconds per note; *P*<.001). These findings demonstrate that quantum-enhanced optimization provides substantial improvements in anonymization quality, particularly reducing FP rates and improving entity-level predictions.

One of the important observations is the precision improvement achieved by quantum-enhanced models. For the NAME entity, Dynex-QML-70B achieved a precision of 0.971 compared to 0.624 for the stand-alone Llama-70B, driven primarily by reduction in FP (10 vs 202). Similar trends were observed for DATE (precision 0.960 vs 0.919) and LOCAL (precision 0.919 vs 0.873). These improvements suggest that the QUBO-based optimization layer acts as an effective corrective mechanism constraining the model’s tendency to overidentify borderline entities, a known limitation of prompt-driven LLM anonymization approaches [28]. By imposing structured optimization criteria, the quantum-enhanced framework appears to reduce overgeneralization while preserving high recall.

A key consideration in clinical anonymization is that sensitivity and precision do not have equivalent practical implications. FN correspond to missed protected health information and therefore represent the primary privacy and regulatory risk. In contrast, FP correspond to overanonymization, which may reduce the clinical interpretability and downstream research utility of the resulting text. For this reason, the performance gain observed with Dynex-QML (Llama 70B) should not be interpreted as a simple precision improvement. Rather, the relevant finding is that the model achieved higher precision while preserving high sensitivity across most entity categories. This pattern is illustrated in Figure 2, where Dynex-QML (Llama 70B) occupies the most favorable region of the macro-sensitivity and macro-precision space, and in Figure 3, where the reduction in FP is not accompanied by a meaningful increase in FN.

Relative performance across entity categories also highlights persistent challenges in clinical text anonymization. All models achieved their strongest results on DATE entities, which is expected given their relatively standardized formats and limited contextual ambiguity in clinical narratives. In contrast, the ID category remained the most difficult to anonymize reliably; even the highest-performing model (Dynex-QML-70B) reached only a 0.548 *F*_1_-score. This limitation likely reflects the substantial variability in identifier formats across institutions, specialties, and documentation contexts, as well as the inherent difficulty in distinguishing clinically relevant numerical values from patient-specific identifiers.

The interannotator agreement analysis provides important context for interpreting the model results. The overall mean pairwise entity-level macro*–F*_1_-score of 0.786 (SD 0.038) indicates generally consistent application of the annotation guidelines, although agreement varied across entity categories. This variability suggests that some model errors may reflect both model limitations and uncertainty in the reference annotations. However, all models were evaluated against the same operational gold standard, and the improvements achieved by Dynex-QML 70B were observed across multiple entity categories. The lower and less precise agreement observed for ID should be interpreted cautiously because this category was represented by very few entities in the reannotated subset.

### Comparison With Prior Work

Our findings align with extended recent work on LLM-based deidentification. Previous work [28] reported that Llama-3-70B achieved over 0.992 sensitivity on German clinical letters, consistent with our observation of high sensitivity rates (0.951‐0.990) for the larger models. However, their work did not address FP problems that we found to be substantially mitigated by quantum-enhanced optimization.

The performance levels achieved in our study are somewhat lower than those reported on the English i2b2 benchmarks, where top systems achieve a micro-averaged *F*_1_-score of 0.940 [39]. This difference reflects several factors: the Portuguese language context introduces different linguistic challenges; our entity definitions may be broader than i2b2 categories; and prompt-based approaches generally show lower performance than fine-tuned models [26]. Nevertheless, relative improvements from quantum enhancement should generalize across contexts.

In the quantum computing domain, our work represents one of the first practical applications of quantum-enhanced methods to clinical NLP tasks. Prior work on QNLP has focused primarily on theoretical analyses and simple classification tasks [33,40]. The success of our QUBO-based approach suggests that quantum optimization can provide practical benefits even with current hybrid quantum-classical architectures.

### Computational Efficiency Considerations

From an operational standpoint, Dynex-QML-70B showed a lower total elapsed time than Llama-70B (7.97, SD 2.87 seconds per note vs 8.52, SD 3.32 seconds per note). This finding is notable because a quantum-enhanced approach involves additional optimization steps that might be expected to increase computational overhead. The efficiency gains may result from the global optimization reducing the need for multiple inference passes or from the QUBO solver converging faster than iterative token generation in challenging cases.

For smaller models, computational trade-offs are less favorable for the quantum-enhanced approach. Dynex-QML-8B requires substantially more processing time than Llama-8B (8.59, SD 3.62 seconds per note vs 5.22, SD 2.12 seconds per note), suggesting that the overhead of quantum optimization may be more significant relative to the faster base model inference. This makes the 70B configuration more suitable for production deployment where quality is prioritized over raw speed.

### Regulatory Compliance Implications

Improvements in precision achieved by quantum-enhanced approaches have important implications for regulatory compliance. Under HIPAA Safe Harbor provisions, all 18 categories of identifiers must be removed with no residual information that could enable reidentification [11]. High FN rates (missed entities) create direct compliance risks, while high FP rates (overanonymization) can degrade data utility for research purposes.

The Dynex-QML-70B model’s combination of high recall (minimizing compliance risk) and high precision (preserving data utility) represents an optimal balance for health care applications. For sensitive entity types such as names, the model’s 0.951 recall ensures that very few identifying names remain in anonymized output, while the 0.971 precision ensures that clinical terms are not erroneously removed.

### Limitations

Several limitations of this study should be acknowledged. First, our evaluation was conducted using Portuguese outpatient clinical notes obtained within a single health care system. InCor is part of HC-FMUSP, the largest academic health care complex in Latin America, serving a large and diverse patient population through multiple clinical pathways, different documentation styles, clinical specialties, and languages. Institutional differences in writing patterns, abbreviation usage, entity distributions, and documentation workflows may substantially affect anonymization performance in external settings. Second, the dataset size of 1000 texts, while sufficient for comparative evaluation, may not capture the full variability of clinical documentation. Third, although the same 100 randomly selected notes were independently reannotated by 3 annotators, the agreement analysis covered only 20% of the evaluation set and 10% of the complete corpus. It also included 3 of the 5 original annotators rather than the complete annotation team. Fourth, our implementation of quantum-enhanced optimization uses simulated quantum annealing on GPU hardware rather than actual quantum processors, though this reflects the current state of accessible quantum computing resources and the practical deployment model of the Dynex platform.

Additionally, we did not conduct systematic hyperparameter optimization for either the prompt design or the QUBO formulation parameters, suggesting that further improvements may be achievable. Energy consumption measurements were not included in this study due to methodological challenges in accurately comparing energy usage between classical LLM inference and hybrid quantum-classical systems. The Dynex platform’s use of algorithmic qubits emulated in GPU VRAM introduces complexity in energy accounting, as the quantum annealing process involves intricate patterns of memory access and computation that are not straightforwardly comparable to standard neural network inference. Specifically, the algorithmic qubits are not isolated physical devices whose draw can be metered, but mathematical state variables coresident in GPU video memory alongside the classical LLM inference workload, and the Dynex platform operates on a distributed GPU network where node-level power readings are confounded by other tenant workloads sharing the same hardware. We therefore took the methodologically conservative position of removing the energy comparison from this paper rather than reporting a comparison whose denominator is not well defined. Future work should develop standardized methodologies for energy measurement in neuromorphic quantum computing systems to enable fair comparisons with classical approaches.

### Future Directions

Future research should extend this evaluation to additional languages and clinical domains, including radiology reports, pathology notes, and nursing documentation. Systematic benchmarking against fine-tuned NER models and other state-of-the-art systems would provide additional context for the quantum-enhanced approach. As quantum hardware continues to evolve, evaluation on alternative quantum computing platforms would help characterize the generalizability of the QUBO-based anonymization approach.

The QUBO formulation approach may also be applicable to other clinical NLP tasks where global optimization could improve upon sequential processing, such as relation extraction, temporal reasoning, and clinical summarization. Exploring these applications represents a promising direction for quantum-enhanced health care AI. Additionally, investigating the interpretability of the quantum optimization process could provide insights into which linguistic features most strongly influence anonymization decisions.

### Conclusions

This study demonstrates that quantum-enhanced hybrid architectures can achieve substantial improvements in medical text anonymization compared to stand-alone LLMs. The Dynex-QML-70B model achieved the highest overall performance (macro*–F*_1_-score 0.855, macro-precision 0.794, and macro-sensitivity 0.971) while slightly reducing processing time compared to Llama-3.3-70B (7.97, SD 2.87 seconds per note vs 8.52 SD 3.32 seconds per note). The primary advantage of quantum enhancement lies in substantially improved precision by 25% (0.794 for Dynex-QML-70B vs 0.636 for Llama-3.3-70B) while maintaining high sensitivity with a 2% increase (0.971 for Dynex-QML-70B vs 0.949 for Llama-3.3-70B).

The detailed exposition of the quantum-enhanced methodology demonstrates how transformer attention mechanisms can be reformulated as QUBO problems and solved via neuromorphic quantum annealing. This approach leverages the global optimization capabilities of quantum computing to address the precision challenges inherent in prompt-based LLM anonymization.

These findings highlight the growing maturity of hybrid quantum-classical architectures and their promise for accurate and scalable anonymization in real-world health care applications. As regulatory requirements for health data protection continue to evolve and the volume of clinical text data grows, quantum-enhanced approaches may represent a viable path toward sustainable solutions for clinical text anonymization that balance quality, efficiency, and compliance.

## Abbreviations

BERT: Bidirectional Encoder Representations from Transformers
EHR: electronic health record
FN: false negative
FP: false positive
GCSP: Global Constraint Satisfaction Problem
GDPR: General Data Protection Regulation
GPU: Graphics Processing Unit
HC-FMUSP: Hospital das Clínicas of the University of São Paulo Medical School
HIPAA: Health Insurance Portability and Accountability Act
IBGE: Instituto Brasileiro de Geografia e Estatística
*ICD-10*: *International Statistical Classification of Diseases, Tenth Revision*
InCor: Instituto do Coração
LGPD: Lei Geral de Proteção de Dados Pessoais
LLM: large language model
NER: Named Entity Recognition
NLP: natural language processing
ODE: ordinary differential equation
PHI: Protected Health Information
QNLP: Quantum Natural Language Processing
QUBO: Quadratic Unconstrained Binary Optimization
SUS: Sistema Único de Saúde
TI: internal processing time
TP: true positive
TT: total elapsed time
VRAM: video random access memory
